# PDGF-D Expression Is Down-Regulated by TGFβ in Fibroblasts

**DOI:** 10.1371/journal.pone.0108656

**Published:** 2014-10-03

**Authors:** Saima Charni Chaabane, Alexandra Coomans de Brachène, Ahmed Essaghir, Amélie Velghe, Sandra Lo Re, Julie Stockis, Sophie Lucas, Levon M. Khachigian, François Huaux, Jean-Baptiste Demoulin

**Affiliations:** 1 de Duve Institute, Université catholique de Louvain, Brussels, Belgium; 2 Louvain center of Toxicology and Applied Pharmacology, Institute of Experimental and Clinical Research, Université catholique de Louvain, Brussels, Belgium; 3 Walloon Excellence in Life Sciences and Biotechnology (WELBIO), Wallonia, Belgium; 4 Center of Vascular Research, University of New South Wales, Sydney, Australia; University of Nebraska Medical Center, United States of America

## Abstract

Transforming growth factor-β (TGFβ) is a key mediator of fibrogenesis. TGFβ is overexpressed and activated in fibrotic diseases, regulates fibroblast differentiation into myofibroblasts and induces extracellular matrix deposition. Platelet-derived growth factor (PDGF) is also a regulator of fibrogenesis. Some studies showed a link between TGFβ and PDGF in certain fibrotic diseases. TGFβ induces PDGF receptor alpha expression in scleroderma fibroblasts. PDGF-C and -D are the most recently discovered ligands and also play a role in fibrosis. In this study, we report the first link between TGFβ and PDGF-D and -C ligands. In normal fibroblasts, TGFβ down-regulated PDGF-D expression and up-regulated PDGF-C expression at the mRNA and protein levels. This phenomenon is not limited to TGFβ since other growth factors implicated in fibrosis, such as FGF, EGF and PDGF-B, also regulated PDGF-D and PDGF-C expression. Among different kinase inhibitors, only TGFβ receptor inhibitors and the IκB kinase (IKK) inhibitor BMS-345541 blocked the effect of TGFβ. However, activation of the classical NF-κB pathway was not involved. Interestingly, in a model of lung fibrosis induced by either bleomycin or silica, PDGF-D was down-regulated, which correlates with the production of TGFβ and other fibrotic growth factors. In conclusion, the down-regulation of PDGF-D by TGFβ and other growth factors may serve as a negative feedback in the network of cytokines that control fibrosis.

## Introduction

Transforming growth factor-β (TGFβ) family members are cytokines that regulate numerous physiological processes such as embryonic development, cell differentiation, proliferation, migration and extracellular matrix production [1], [2]. TGFβ has also been linked to several human pathologies including fibrosis and carcinogenesis [3], [4], [5]. TGFβ is a central mediator of several fibrotic diseases like renal fibrosis or liver fibrosis [6], [7]. Smad proteins are the core components of the intracellular signaling cascade of TGFβ receptors. The Smad proteins transduce signals from the cell surface directly to the nucleus where they regulate gene transcription in cooperation with co-activators and/or co-repressors. In addition to the Smad pathway, also known as the conventional pathway, Smad-independent pathways can be activated by TGFβ, including TGFβ-associated kinase 1 (TAK1) [8], certain MAPKs such as extracellular signal-regulated kinase (ERK) 1 and 2, Jun N-terminal kinase (JNK) and p38 mitogen-activated protein kinase [8], [9]. The PI3K-Akt-mTOR pathway has also been reported to be activated by TGFβ [10]. The importance of these alternative pathways, NF-κB in particular, has been debated. NF-κB activation by TGFβ seems to be cell type dependent [11], [12].

Platelet-derived growth factors (PDGF) are also major players in fibrosis including intestinal, cardiac and liver fibrosis [13], [14], [15]. PDGFs are potent mitogens for myofibroblasts while TGFβ stimulates collagen deposition by these cells [16], [17]. PDGF family members are composed of 4 ligands A, B, C and D binding to two receptors, alpha and beta. PDGF-A and -B are well characterized and their role in fibrotic diseases is well established [13], [14], [15]. PDGF-C and -D were discovered more recently and are also implicated in fibrosis. PDGF-C is overexpressed and is a potent inducer of fibrosis in the heart [18], lung [19], kidney [20], pancreas [21] and liver [22]. Fewer studies have linked PDGF-D to fibrotic diseases. PDGF-D is overexpressed in fibrosis of the liver [23] and kidney [24]. By contrast, one study showed a negative correlation of PDGF-D expression in a mice model of bleomycin-induced lung fibrosis [19].

Crosstalk between TGFβ and PDGF has been reported in several fibrotic conditions. In a model of hepatic fibrosis, one report showed that TGFβ mediated the mitogenic effect of PDGF-BB, but not PDGF-AA, and this effect was due to the up-regulation of PDGFRβ mRNA and cell surface expression [25]. In scleroderma, TGFβ also enhances PDGF-A-stimulated growth by increasing the expression of PDGFRα [26]. All together these are the most relevant studies that reported a regulation of PDGFs by TGFβ in fibrotic disorders.

In the present study, we demonstrate that TGFβ down-regulates PDGF-D expression in normal fibroblasts.

## Materials and Methods

### Ethics Statement

This study was approved by the local ethical committee for animal experiments (Comité d'Ethique pour l'Expérimentation Animale, Permit number LA1230312). Experiments were carried out in strict accordance with the Belgian and European regulations. All surgery was performed after euthanasia by intraperitoneal injection of 20 mg of sodium pentobarbital (Certa, Braine l'Alleud, Belgium), and all efforts were made to minimize suffering.

### Reagents and antibodies

Sources were as follows: Recombinant human TGFβ1, PDGF-BB, EGF, FGF-4 and IL1β were purchased from Peprotech; p38 inhibitors SB202190 and SB203580 from Millipore; HDAC inhibitor Trichostatin (TSA) was a kind gift from Pr. De Smet (Université catholique de Louvain, Belgium); ALK5 inhibitor SB431542, IKK inhibitor BMS-345541 and MEK1/2 inhibitor U0126 were from Calbiochem; PI3K inhibitor LY294002 from Cayman chemical; JNK inhibitor SP600125 from Sigma, PDGF-D antibody from Santa Cruz; PDGF-C monoclonal antibody was a kind gift from Pr. U. Eriksson (Ludwig Institute for Cancer Research & Karolinska Institute, Sweden) [27]. Actin antibody was from Sigma; anti-mouse HRP from Santa Cruz.

### Cell lines

MRC5 human lung fibroblasts (Sigma-Aldrich), AG01518 and AG01523 human foreskin fibroblasts (both from Coriell Institute for Medical Research, Camden, NJ) [28] were grown in Quantum 333 fibroblast growth medium (PAA laboratories, Pasching, Austria). HEK293T (from ATCC) cells were grown in DMEM supplemented with 10% fetal bovine serum (FBS), 50 U/ml penicillin and 50 µg/ml streptomycin (Gibco, Life Technologies). Gamma2A human fibrosarcoma cells (a kind gift from Prof Stefan Constantinescu, de Duve Institute, Belgium) were grown as described by Dusa et al. [29].

### Lentivirus-mediated stable gene silencing

Short hairpin RNA (shRNA) used in this study were as follows: (**i**) pLKO.1 scramble shRNA: CCTAAGGTTAAGTCGCCCTCG (Addgene, 1864), (**ii**) pGIPZ shsmad4 (1): AGCAGTGACTTTGTATAGAGAA (Open Biosystems, V2LHS_37199), (**iii**) pGIPZ shsmad4 (2): CACTGCTAAATTCTATGTTAAA (Open Biosystems, V2LH6_37196). Lentiviral plasmids expressing the above shRNAs were transfected with the packaging plasmid pCMV-dr8.2 dvpr and the envelope plasmid pCMV-VSV-G (Addgene catalog numbers 8455 and 8454) into HEK293T cells using FuGENE 6 (Roche Applied Science) as described [30]. Virus-containing supernatants were collected at 36 and 60 hours after transfection and used to infect fibroblasts in the presence of 8 µg/ml of polybrene (Sigma). Then cells were incubated with standard growth medium containing 2 µg/ml puromycin for 3 days prior to cell analysis.

### Reverse transcriptase-PCR and quantitative PCR analysis

Subconfluent AG01523 or MRC5 fibroblasts were plated at 600000 cells/plate, starved for 24 hours in the presence of 0.05% essentially fatty acid free BSA (Sigma) and treated for 30 minutes in presence or absence of the following inhibitors: TGFβ receptor inhibitor (SB431542, 10 µM), MEK1/2 inhibitor (U0126, 10 µM), p38 inhibitors (SB203580 and SB202190, 10 µM), IKK inhibitor (BMS-345541, 10 µM), PI3K inhibitor (LY294002, 20 µM), HDAC inhibitor (TSA, 300 nM) or vehicle (DMSO). Cells were then incubated during 24 hours in the presence of TGFβ (4 ng/ml). Total RNA was extracted from cells using the Qiagen RNeasy extraction kit according to the manufacturer's instructions. Complementary DNA synthesis from total RNA was performed following the M-MLV reverse transcriptase (Invitrogen) protocol. Real time quantitative PCR (q-PCR) was performed using SYBR-green (ABgene kit) on an iCycler instrument (Bio-Rad) as described [30]. The expression of the RPLP0 and β-Actin genes was used to normalize q-PCR experiments. All q-PCR primer sequences used in this study are provided in Table S1.

### Protein extraction and Western blotting

Subconfluent MRC5 cells were plated at 200000 cells/well (six well plates), starved for 24 hours in the presence of 0.05% essentially fatty acid free BSA (Sigma) and treated with or without TGFβ (4 ng/ml) during 24 or 48 hours. Cells were washed with phosphate-buffered saline (PBS) and then lysed as described [30]. Equal amounts of total protein extracts were analyzed by SDS-PAGE, then transferred on a polyvinylidene difluoride membrane (PVDF) and subjected to antibodies against PDGF-D (1 µg/ml, Santa Cruz), PDGF-C (1 µg/ml, monoclonal antibody) or β-actin (0.5 µg/ml, Sigma).

### Luciferase assay

Gamma-2A cells were plated in 24-well plates and transfected using Lipofectamine 2000 (Invitrogen) with 1 µg p1168-PDGF-D-luc vector [31] or 1 µg of CAGA12 vector [32] as a control, 1 µg pcDNA3 or pEF-BOS-Alk5 [33] and 1.5 µg pE1F-β-galactosidase (pDRIVEchEF1-RU5, Invitrogen) [28]. Cells were incubated for 4 hours, washed with PBS, and then cultured in normal medium for 24 hours. Cells were then treated with 10 µM of IKK inhibitor (BMS-345541) 30 min prior to TGFβ stimulation (4 ng/ml) for 24 hours. We then measured the luciferase and β-galactosidase activities.

### Enzyme-linked immunoabsorbent assays (ELISA)

An ELISA kit was obtained to measure concentrations of PDGF-DD (R&D systems) in supernatants of MRC5 cells cultured in the absence or presence of TGFβ (4 ng/ml) for 24 h. The detection limit of this assay was 1.67 pg/ml.

### Lung Fibrosis induced with crystalline silica particles

NMRI mice were instilled either with 2.5 mg of silica per mouse or with NaCl for control condition. Three mice were used for control group and four for the silica-treated group. Mice were sacrificed 60 or 120 days post instillation by intraperitoneal injection of 20 mg of sodium pentobarbital (Certa, Braine l'Alleud, Belgium) and total RNA was extracted from their lung samples [34].

### Statistical analysis

Results were analyzed by Student's t-test. Multiple comparisons were carried out using analysis of variance (ANOVA test). A significance threshold was set on p-values ≤0.05. All experiments were performed in at least three independent replicates.

## Results

### PDGF-D and PDGF-C are regulated by TGFβ at the mRNA and protein levels

**Figure 1:**
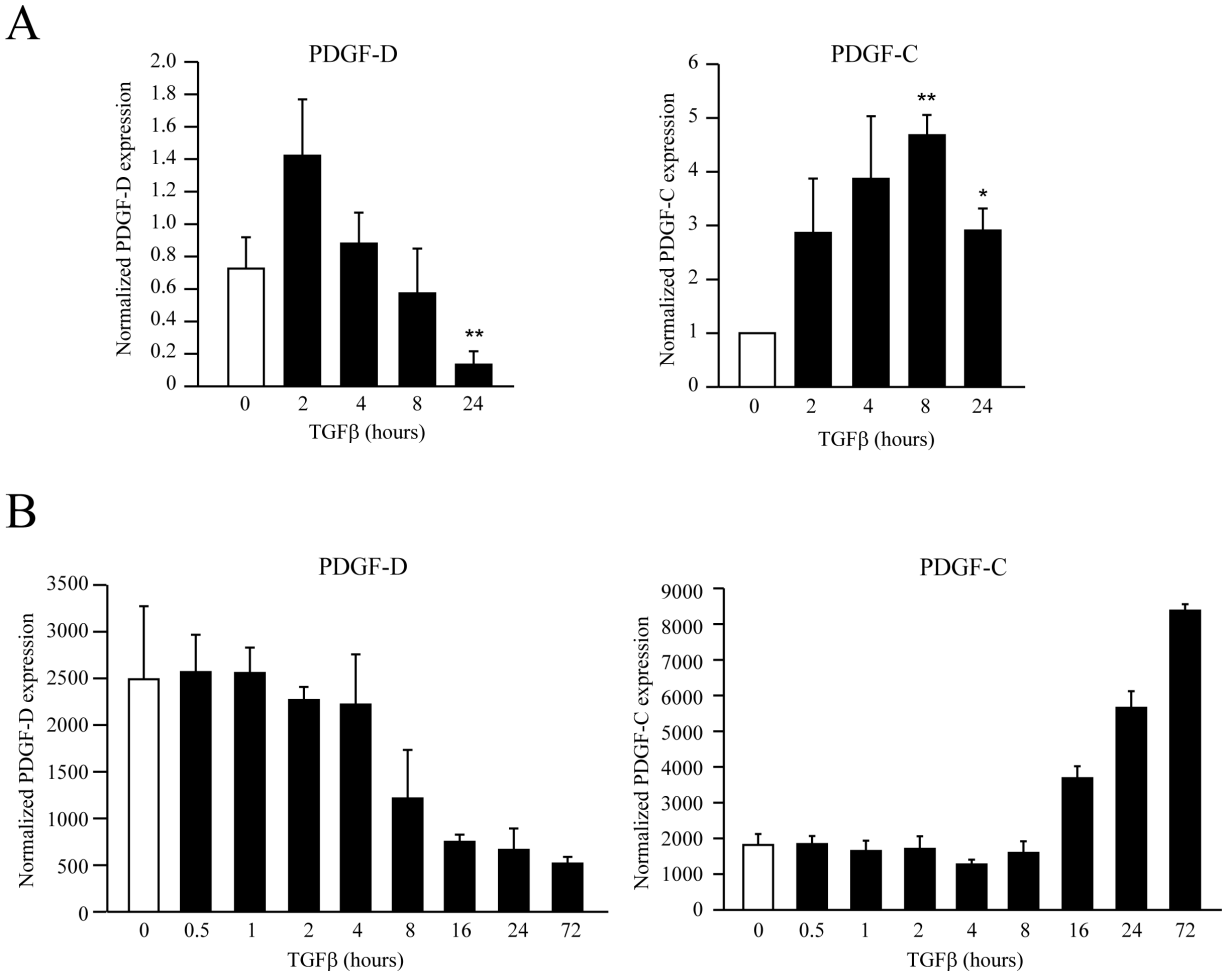
PDGF-D and PDGF-C are late targets of TGFβ. (A) MRC5 human fibroblasts were treated with TGFβ (4 ng/ml) for 2 to 24 hours. PDGF-D and PDGF-C mRNA expression was analyzed by RT-qPCR and normalized with the housekeeping gene RPLP0 (*, p<0.05; **, p<0.01). (B) Published microarray data were also reanalyzed for PDGF-C and –D expression (GEO database, reference GDS3710). In this experiment, A549 cells were stimulated by TGFβ1 for the indicated periods of time. Each time point was investigated in triplicates (p<0.001, ANOVA).

**Figure 2:**
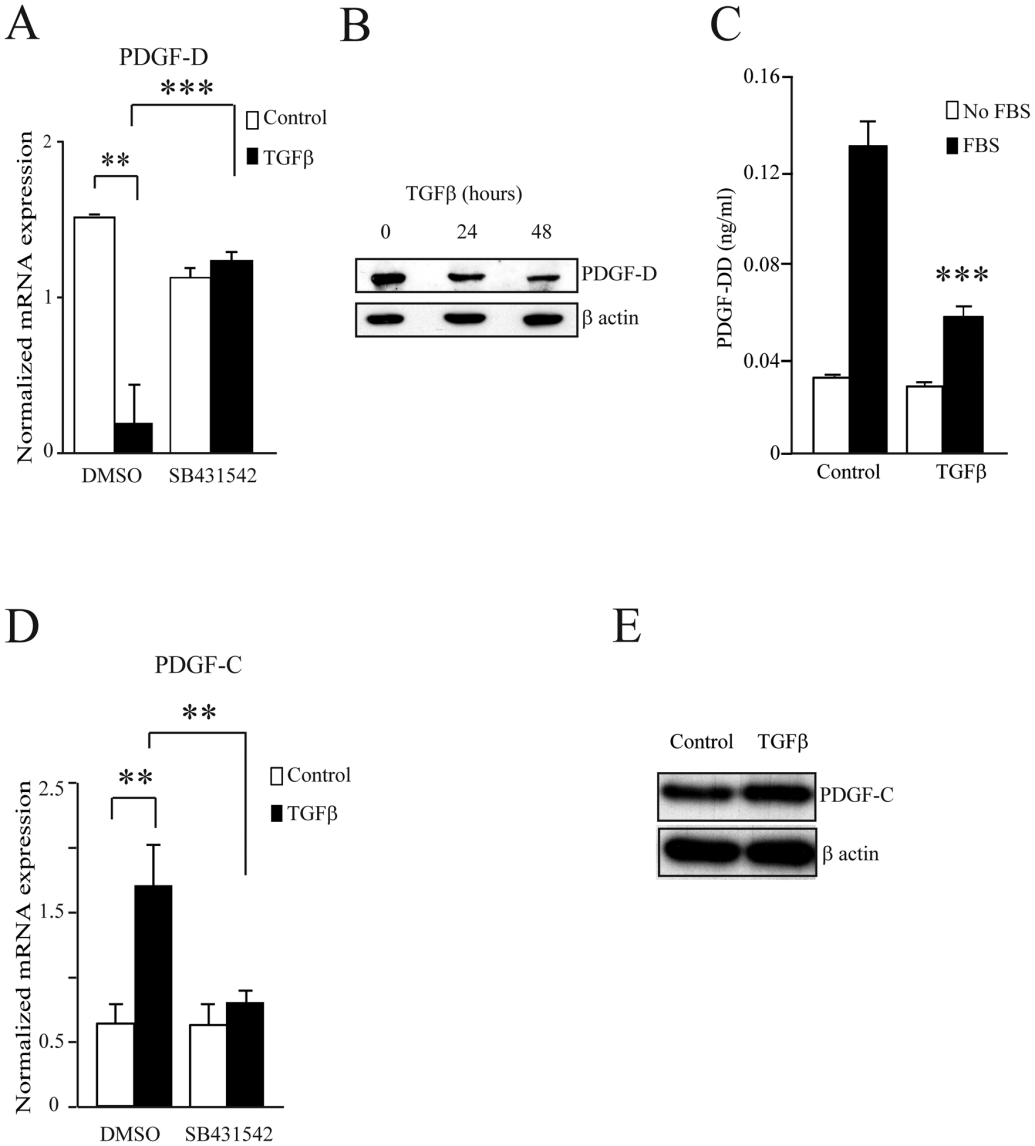
PDGF-D and PDGF-C regulation by TGFβ at the mRNA and protein levels. (A) and (D), MRC5 human fibroblasts were pre-treated with TGFβ receptor inhibitor SB431542 (10 µM) 30 min before treatment with TGFβ (4 ng/ml) for 24 h. The control condition was treated with DMSO alone. PDGF-D and PDGF-C mRNA expression was measured by RT-qPCR and normalized with the housekeeping gene RPLP0. (B) and (E) MRC5 cells were treated with TGFβ (4 ng/ml) for the indicated period of time. PDGF-D and PDGF-C protein expression was analyzed by western blotting. Blots were re-hybridized with an anti-β-actin antibody as control. (C) Active PDGF-DD levels were measured by ELISA in the culture supernatant of MRC5 cells treated or untreated with TGFβ 4 ng/ml for 24 h. The average of 3 experiments is shown with S.E.M, ** p<0.005, *** p<0.0005.

We analyzed the expression of PDGF-D and PDGF-C genes by RT-qPCR in MRC5 lung fibroblasts treated or not with TGFβ for various periods of time. PDGF-D mRNA expression was significantly down-regulated by TGFβ, whereas PDGF-C mRNA was up-regulated (Figure 1A). We also observed a similar regulation of these genes by TGFβ in AG01518 and AG01523 fibroblasts (data not shown). By reanalyzing a publicly available time course experiment, in which A549 epithelial cells were stimulated with TGFβ during 72 hours (GEO, reference GDS3710), we observed that, starting from 16 hours of TGFβ stimulation, PDGF-D and PDGF-C were strikingly regulated in an opposite manner, as in MRC5 cells (Figure 1B). To investigate whether these observations were specific to TGFβ we treated the MRC5 cells with a selective inhibitor of TGFβ receptor activity (SB431542, Figures 2A and 2D). As expected, the inhibitor reversed the effects of TGFβ on PDGF-C and PDGF-D expression levels.

PDGF-C and PDGF-D regulation by TGFβ was confirmed at the protein level by western blotting (Figures 2B and 2E). We next measured PDGF-DD secretion by ELISA, using antibodies that recognize the active form of PDGF-DD, in which the CUB domain was removed. TGFβ-induced down-regulation of PDGF-D mRNA expression had a significant effect on its secretion in the culture medium (Figure 2C). This was reflected by the lower levels of active PDGF-DD protein in the supernatant following exposure of the cells to TGFβ in the presence of serum. We did not see any effect of TGFβ in the absence of serum, most likely because it provides plasmin, which cleaves latent PDGF-DD [35].

### Role of Smad4 in the down-regulation of PDGF-D by TGFβ

To further investigate the mechanism behind PDGF-D down-regulation by TGFβ stimulation, we tested the involvement of the Smad pathway. We infected MRC5 fibroblasts with two different shRNAs against Smad4 and compared PDGF-C and -D mRNA expression after 24 hours in TGFβ-treated versus non-treated cells. TGFβ strongly decreased PDGF-D expression, and this reduction was only marginally prevented by Smad4 knock-down (p<0.05; Figure S1A). The shRNA did not affect PDGF-C regulation by TGFβ (Figure S1B). As a control, we investigated Collagen1A1 (Col1A1) expression, a well-known target of the TGFβ-Smad pathway [36]. TGFβ treatment induced Col1A1 mRNA expression, and this induction was partially decreased by Smad4 shRNA (Figure S1C). This partial effect can be explained by the fact that Smad4 expression was not completely abolished (Figure S1D).

### Role of Smad-independent pathways in PDGF-D gene regulation by TGFβ

**Figure 3:**
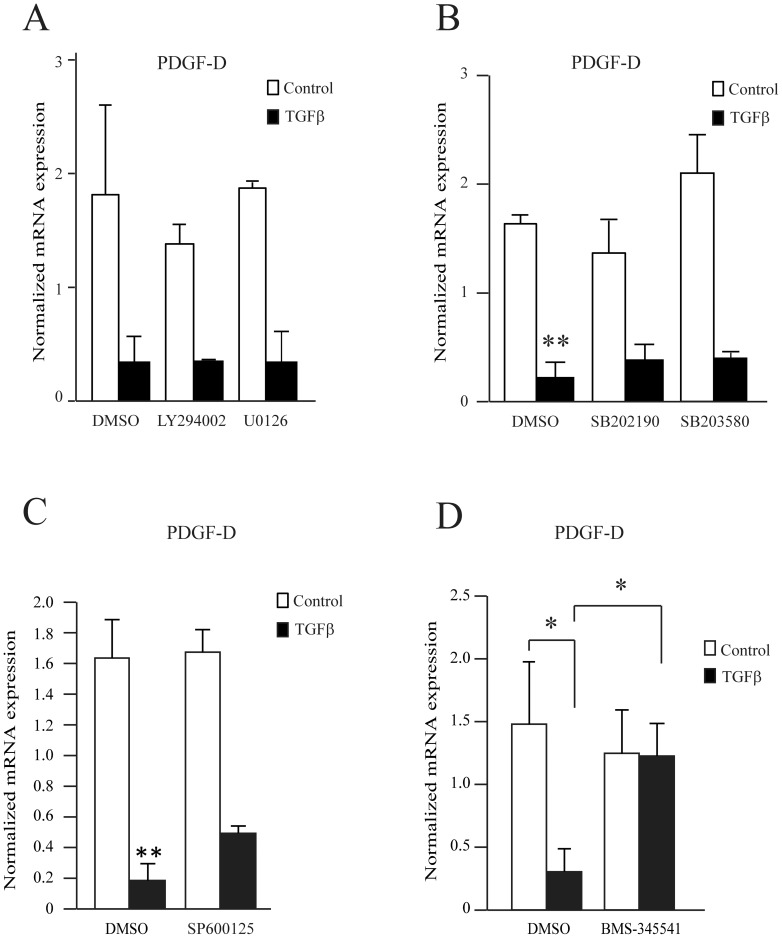
PDGF-D is regulated in a non-conventional manner by TGFβ. MRC5 cells were pre-treated with (A) the PI3K inhibitor LY294002 20 µM or the MAPK inhibitor U0126 10 µM, (B) with an inhibitor of p38 (SB202190 or SB203580, 10 µM), (C) with a JNK inhibitor SP600125, 20 µM and (D) with the IKK inhibitor BMS-345541 10 µM or vehicle (DMSO) for 30 min, then treated or untreated with TGFβ 4 ng/ml for 24 h. PDGF-D mRNA expression was measured by RT-qPCR and normalized with RPLP0 expression. The average of 3 experiments is shown with S.E.M, ** p<0.005, *** p<0.0005.

We next tested whether TGFβ regulates PDGF-D through a Smad-independent pathway. We used specific pharmacological inhibitors to inhibit the PI3K, MAPK and IKK pathways in the presence or absence of TGFβ. Figure 3A shows the effect of PI3K (LY294002) and MEK1/2 (U0126) inhibitors, Figure 3B, the effect of two p38 MAPK inhibitors (SB202190 and SB203580) and Figure 3C, the effect of a JNK inhibitor (SP600125). None of these inhibitors influenced PDGF-D expression. These results suggested that neither the PI3K nor MAPK pathways mediated PDGF-D regulation by TGFβ. We next investigated the NF-κB pathway, by treating fibroblasts with an inhibitor of IKK (BMS-345541). The IKK inhibitor alone had no effect on basal PDGF-D expression, but it abrogated the inhibition of PDGF-D expression observed in the presence of TGFβ (Figure 3D). Similar results were obtained in AG01523 fibroblasts (data not shown). These data suggest that TGFβ regulates PDGF-D via IKK or another unknown BMS-345541 target.

### PDGF-D is regulated at the transcriptional level by TGFβ through IKK

**Figure 4:**
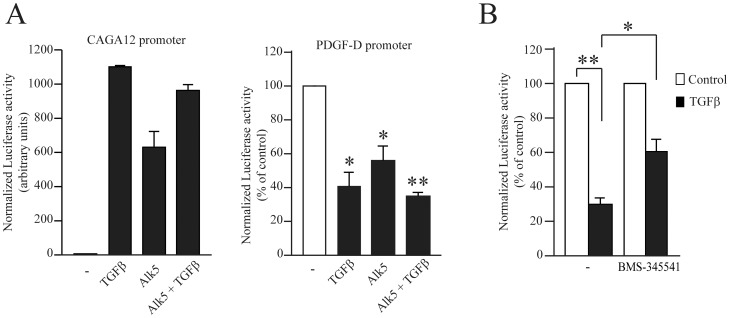
TGFβ represses PDGF-D at the transcriptional level through an IKK-dependent pathway. We used a 1.3 kb fragment of the PDGF-D promoter cloned upstream of the luciferase reporter gene [31]. (A) Gamma-2A cells were co-transfected with the PDGF-D promoter construct or the CAGA12 promoter construct (positive control), with either empty vector or constitutively activated Alk5 [33] and with a control β-galactosidase reporter [28]. Cells were treated with TGFβ 4 ng/ml or left untreated for 24 h. (B) Gamma-2A cells were co-transfected with PDGF-D promoter construct and with β-galactosidase. They were treated with BMS-345541 (10 µM) for 30 min and then TGFβ was added for 24 h. In all experiments, luciferase and β-galactosidase activities were measured and the luciferase activity was normalized by dividing by the β-galactosidase activity. The mean of three independent experiments is shown.

We next evaluated whether TGFβ affected the activity of the PDGF-D gene promoter in luciferase-reporter assays. We used gamma-2A fibrosarcoma cells because they are suitable for the transfection of luciferase reporters and because TGFβ down-regulated PDGF-D mRNA in these cells, as in fibroblasts (data not shown). Cells were transfected with a plasmid encoding luciferase under the control of the PDGF-D promoter, and treated or not with TGFβ. The CAGA12 promoter was used as a control promoter, to verify the responsiveness of transfected cells to added TGFβ (Figure 4A). We observed a significant reduction of PDGF-D promoter activity in the presence of TGFβ (Figure 4A). PDGF-D promoter activity was also significantly down-regulated in cells co-transfected with a constitutively activated mutant of the TGFβ receptor chain ALK5 (activin receptor-like kinase), in the presence or absence of TGFβ (Figure 4A). Moreover, IKK inhibition partially reversed the inhibitory effect of TGFβ on PDGF-D promoter activity (Figure 4B), confirming the results shown in Figure 3.

### PDGF-D is down-regulated in a mouse model of lung fibrosis

**Figure 5:**
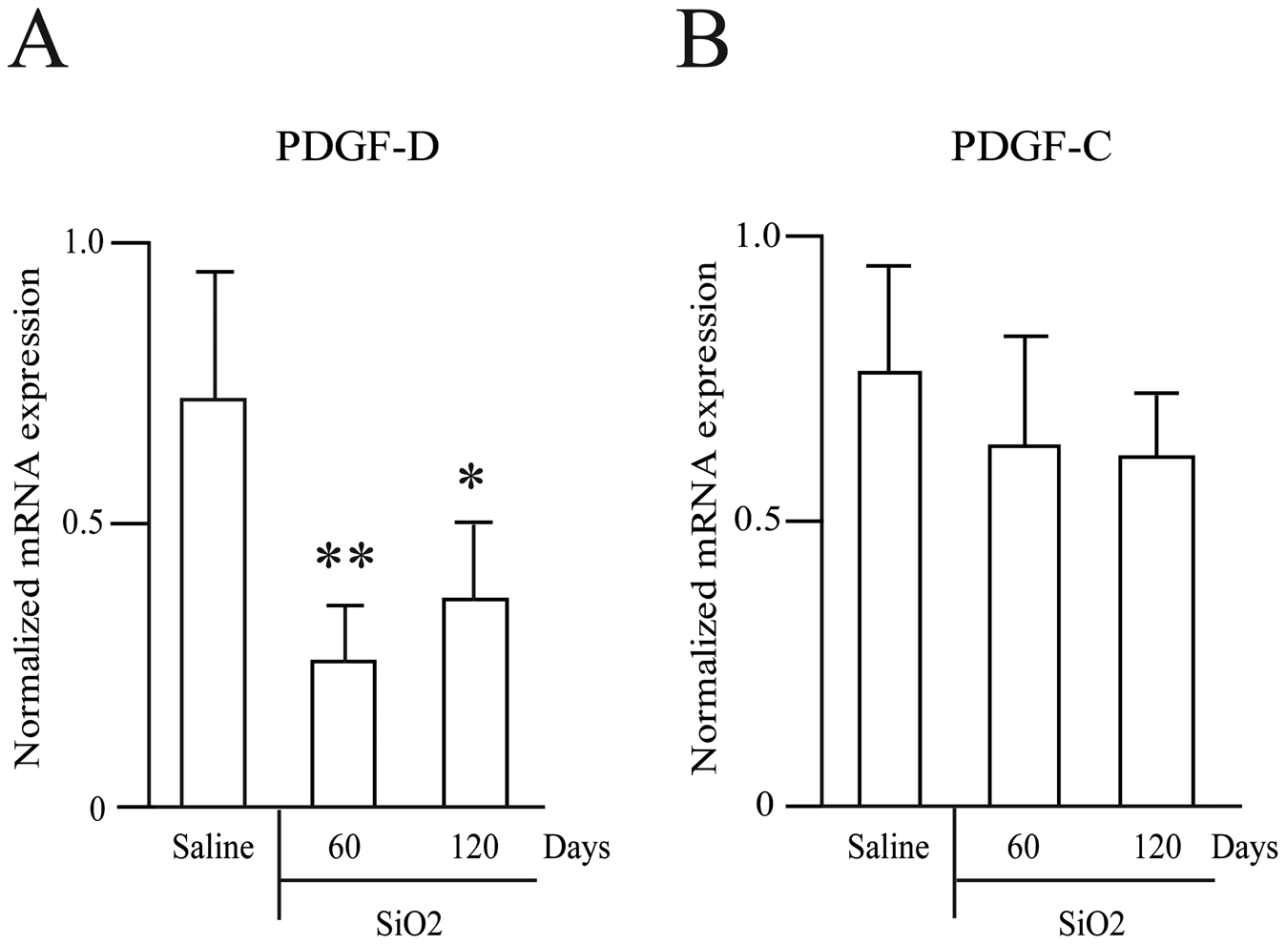
PDGF-D and PDGF-C expression in lung fibrosis induced by silica. RNA samples were harvested from lungs of three control mice and four silica-treated mice. (A) PDGF-D and (B) PDGF-C gene expression was measured by quantitative PCR and reported to the expression of actin. ANOVA analysis revealed that the effect of treatment was highly significant (p-value: <2.2e-16, data not shown).

TGFβ is implicated in several fibrotic diseases. To analyze the expression of PDGF-D and PDGF-C genes in the context of a fibrotic disease, we performed RT-qPCR on lungs collected from NMRI mice that have received intra-tracheal instillations of silica. Mice developed lung fibrosis upon silica instillation, and Rabolli et al. showed that TGFβ is highly expressed and released in fibrotic lungs by comparison to saline controls in this model [34]. PDGF-D and PDGF-C gene expression was analyzed 60 and 120 days post instillation by RT-qPCR. PDGF-D expression was significantly down-regulated in fibrotic lungs by comparison to controls (Figure 5A). PDGF-C expression, however, was not different in fibrotic lungs by comparison to controls (Figure 5B). Similar results were obtained with another strain of mice (C57/Bl6) instilled or not with silica (data not shown).

### PDGF-D is down-regulated by other growth factors involved in fibrosis

**Figure 6:**
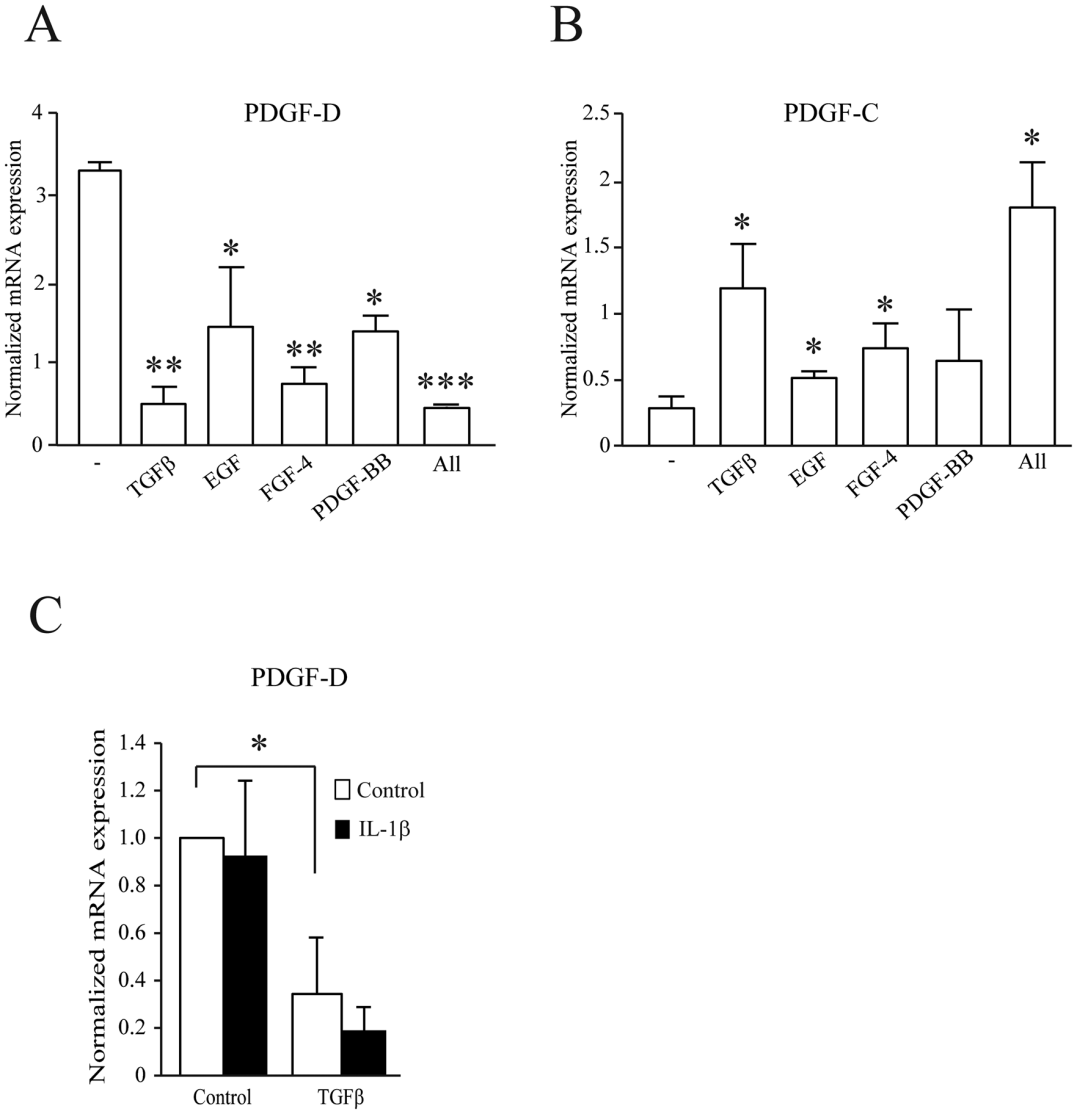
Growth factors regulate PDGF-D and PDGF-C expression. MRC5 cells were treated with TGFβ 4 ng/ml, EGF 50 ng/ml, FGF-4 10 ng/ml (in the presence of heparin 10 µg/ml), PDGF-BB 20 ng/ml alone or all together for 24 h and PDGF-D (A) and PDGF-C (B) gene expression was measured by RT-qPCR. (C) AG01523 fibroblasts were treated with TGFβ 4 ng/ml, IL-1β 10 ng/ml or both for 24 h and PDGF-D gene expression was measured by q-PCR. The average of 3 experiments is shown with S.E.M, * p<0.05, ** p<0.005, *** p<0.0005.

We next tested whether, in addition to TGFβ, other growth factors could also regulate PDGF-D and PDGF-C expression. In our previous microarray analysis, we found that PDGF-D expression was significantly down-regulated in fibroblasts 24 hours after stimulation with PDGF-BB or FGF-2 (Figure S2). Using RT-qPCR, we observed that FGF, EGF and PDGF-BB down-regulated PDGF-D and up-regulated PDGF-C expression (Figures 6A and 6B). We also investigated whether inflammatory cytokines could regulate PDGF-D expression. Interleukin-1β, alone or in combination with TGFβ, did not regulate PDGF-D expression (Figure 6C).

## Discussion

In this study we have shown that TGFβ regulates PDGF-C and -D ligand expression at both the mRNA and the protein level in normal human fibroblasts. To our knowledge, this is the first study showing that the expression of PDGF-C and -D ligands is regulated by TGFβ. Furthermore, a recent study reported that Bone morphogenetic proteins (BMPs), which are members of the of TGFβ superfamily, are repressors of PDGF-A in ovarian granulosa cells and tumors [37]. Here we showed that TGFβ induced PDGF-C in normal fibroblasts whereas PDGF-D was significantly down-regulated at the transcriptional level. Consistently, TGFβ inhibited PDGF-D secretion by the cells.

Smad proteins mediate TGFβ signaling. Down-regulation of Smad4 weakly restored PDGF-D expression in the presence of TGFβ. These partial effects might be explained in part by the fact that Smad4 expression was not completely abolished but Smad-independent pathways are likely to play a role. Additional non-canonical pathways are known to transduce TGFβ signals, and include ERK1/2, p38, JNK or PI3K pathways [10]. We showed that neither the PI3K nor the MAPK pathways are responsible for PDGF-D regulation by TGFβ. Moreover, a study performed in renal fibrosis identified two non-canonical TGFβ pathways, Abl and mTORC1, that play an important role in the development of fibrosis [38]. Our experiments with imatinib (an Abl inhibitor) and rapamycin (an mTOR inhibitor) did not support the hypothesis that these pathways played a role in PDGF-D regulation by TGFβ (data not shown). BMS-3455541, an IKK inhibitor, rescued PDGF-D expression to the basal level in the presence of TGFβ suggesting a regulation through IKK.

Whether TGFβ activates NF-κB pathway is not fully understood and appears to be cell context dependent. Gingery et al. described a mechanism of NF-κB activation by TGFβ through a TAK1/MEK1/2/AKT pathway, which promotes osteoclast survival in cooperation with Smad2/3 [39]. We showed that TGFβ failed to induce p65 phosphorylation on Ser 536 in contrast to the anti-inflammatory cytokine IL-1β which strongly activated p65 (data not shown) but did not regulate PDGF-D in MRC5 fibroblasts. This demonstrates that activation of the classical NF-κB pathway alone does not regulate PDGF-D.

Moreover, an interesting study reported a non-canonical NF-κB activation pathway through TAK1 and NIK by silica in Rat2 fibroblasts [40]. Taken together with our results, it is likely that TGFβ activates NF-κB in fibroblasts through a non-classical pathway. Alternatively, IKK may also signal independently of NF-κB downstream TGFβ. Finally, we cannot rule out a non-specific effect of the IKK inhibitor.

Gene down-regulation frequently involves histone deacetylases (HDAC). Figure S3 showed that HDAC strongly down-regulated PDGF-D expression. Indeed, trichostatin A (TSA), an inhibitor of HDAC, induced PDGF-D expression. However, TGFβ strongly repressed PDGF-D expression even in the presence of HDAC inhibitor, indicating that HDAC regulated PDGF-D independently of TGFβ. Little information is available regarding PDGF-D promoter regulation. Some studies reported that PDGF-D is induced by Ets-1 and Sp1 transcription factors [41]. Sp1 is in general described as an activator rather than a repressor of transcription but some studies reported that Sp1 could act as an inhibitor. Irvine et al. showed that TGFβ inhibits LPL gene transcription through modulation of the action of Sp1 and Sp3. The authors reported that TGFβ stimulation reduces Sp1 DNA binding [42]. Another study suggested also a negative regulation of the AT1 receptor by TGFβ through Sp1.

Unpublished experiments obtained in the lab, demonstrated that the presence of serum down-regulated PDGF-D expression and that this effect was even stronger in the presence of saturating amounts of TGFβ, suggesting that other growth factors may regulate PDGF-D and PDGF-C expression. We showed that EGF, FGF and PDGF-BB alone or combined reduced significantly PDGF-D expression and induced PDGF-C expression confirming our observation with serum. Interestingly, all these growth factors have been linked to fibrosis: PDGF-B in liver fibrosis [15], EGF in heart and renal fibrosis [43], [44] and FGF-4 in lung and renal fibrosis [45], [46]. These factors do not efficiently activate Smad or IKK proteins, thus further experiments will be required to identify the pathway responsible for this effect. Moreover, some studies have reported that FGF represses gene expression via the ERK/Sp1 pathway. Bonello et al. showed in smooth muscle cells that FGF-2 represses PDGFRα expression in an ERK/Sp1 dependent manner. Phosphorylation of Sp1 upon FGF-2 stimulation switches Sp1 from an activator to a repressor of PDGFRα expression [47]. This pathway was also reported in another study in breast cancer cells where the authors showed that FGF-2 down-regulates TSP50 expression via the ERK/Sp1 pathway [48]. All together these studies suggest a mechanism of negative regulation via Sp1 upon FGF-2 activation. Our preliminary results do not go in the same direction as these studies because U0126, an inhibitor of ERK1/2 pathway, did not have any effect on PDGF-D expression upon FGF-4 stimulation (data not shown).

Our observations in cultured fibroblasts may be relevant in pathophysiological conditions. Indeed, we showed in a mouse model of silica-induced lung fibrosis, in which TGFβ expression is highly increased [34], that PDGF-D mRNA expression was down-regulated whereas PDGF-C mRNA expression remained unchanged. These results were confirmed in two different strains of mice. Furthermore, Zhuo et al. have described in another mouse model of lung fibrosis induced by bleomycin that PDGF-D expression was decreased and PDGF-C expression was increased [19]. Our results shed a light on the potential mechanism of PDGF-D regulation in this model of lung fibrosis. However, it is unclear which cytokine regulates PDGF-D in vivo.

In summary, we showed that TGFβ down-regulated PDGF-D and up-regulated PDGF-C expression. The regulation of PDGF-D expression was sensitive to BMS-345541. We speculate that this regulation may represent a negative feedback in the network of fibrotic cytokines.

## Supporting Information

Figure S1 **Role of Smad4 in PDGF-D regulation by TGFβ.**
(TIF)

Figure S2 **PDGF-B and FGF-2 decrease PDGF-D expression.**
(TIF)

Figure S3 **The HDAC inhibitor TSA regulates PDGF-D expression.**
(TIF)

Table S1 **q-PCR primer sequences.**
(DOCX)
