# Supplementary figures and images for: PDGF-D Expression Is Down-Regulated by TGFβ in Fibroblasts
Source: PLoS One. 2014 Oct 3;9(10):e108656. doi: 10.1371/journal.pone.0108656 (PMC4184810; doi:10.1371/journal.pone.0108656)

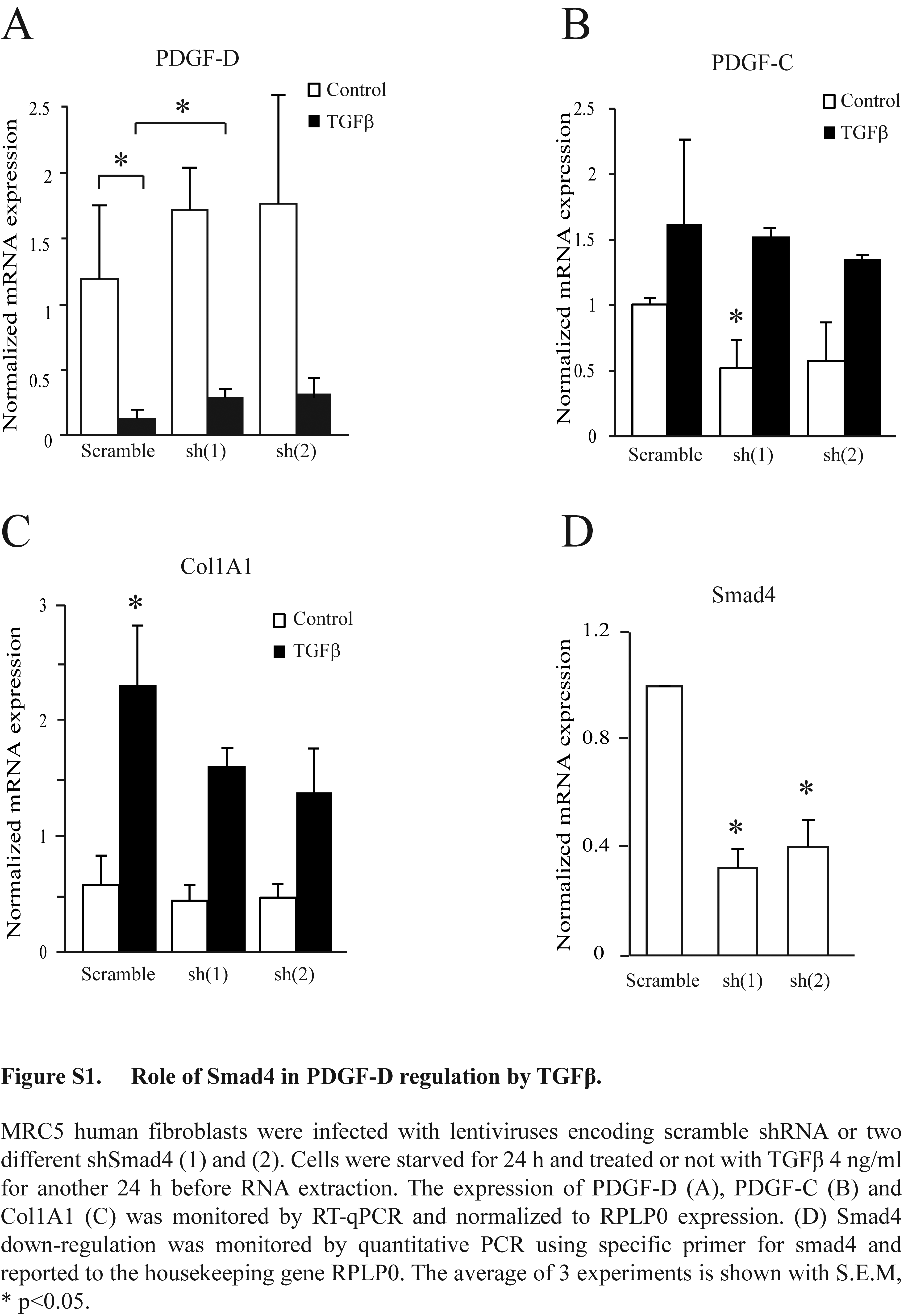

Supplement: Figure S1 — Role of Smad4 in PDGF-D regulation by TGFβ. (TIF) [file pone.0108656.s001.tif]

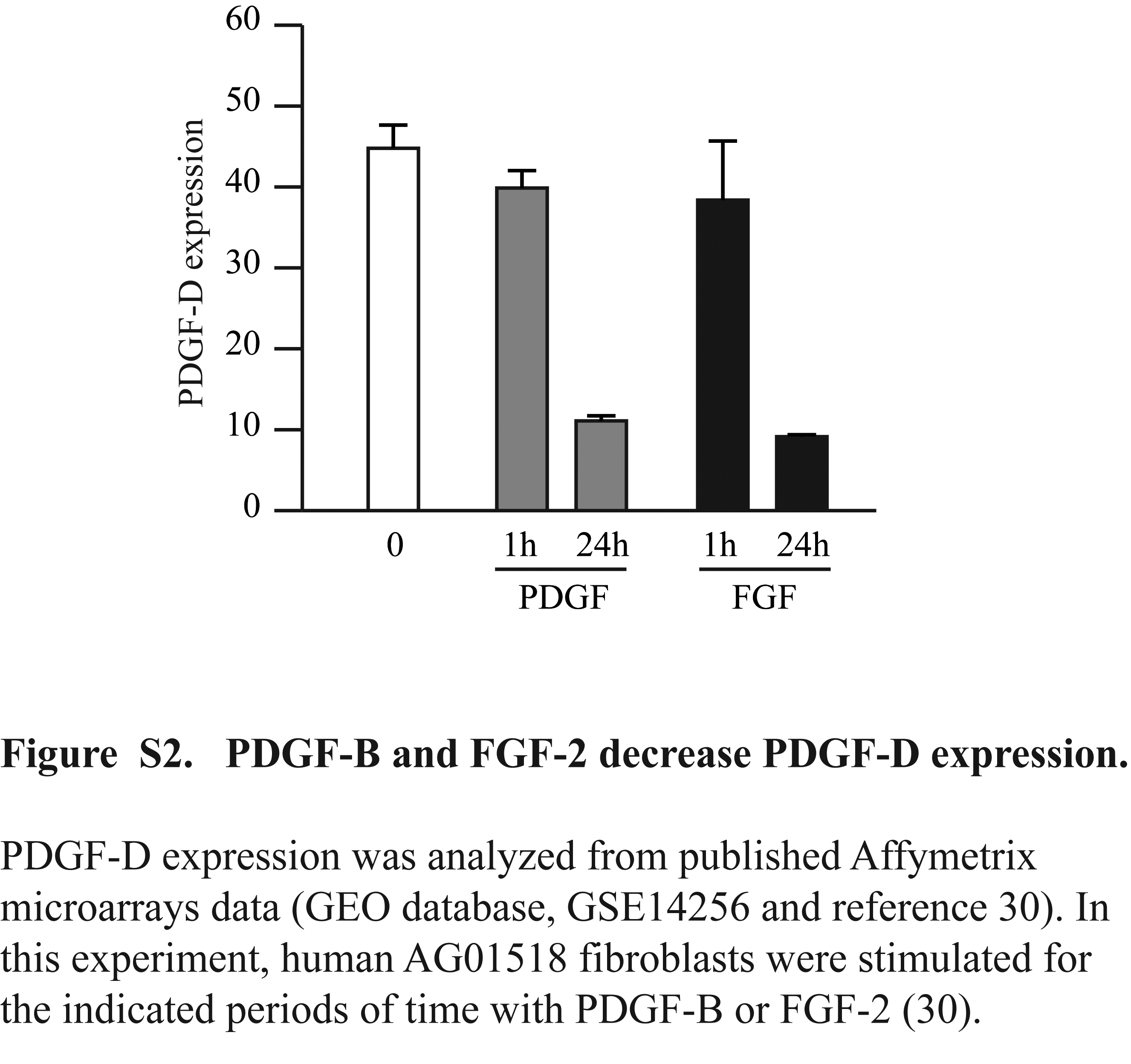

Supplement: Figure S2 — PDGF-B and FGF-2 decrease PDGF-D expression. (TIF) [file pone.0108656.s002.tif]

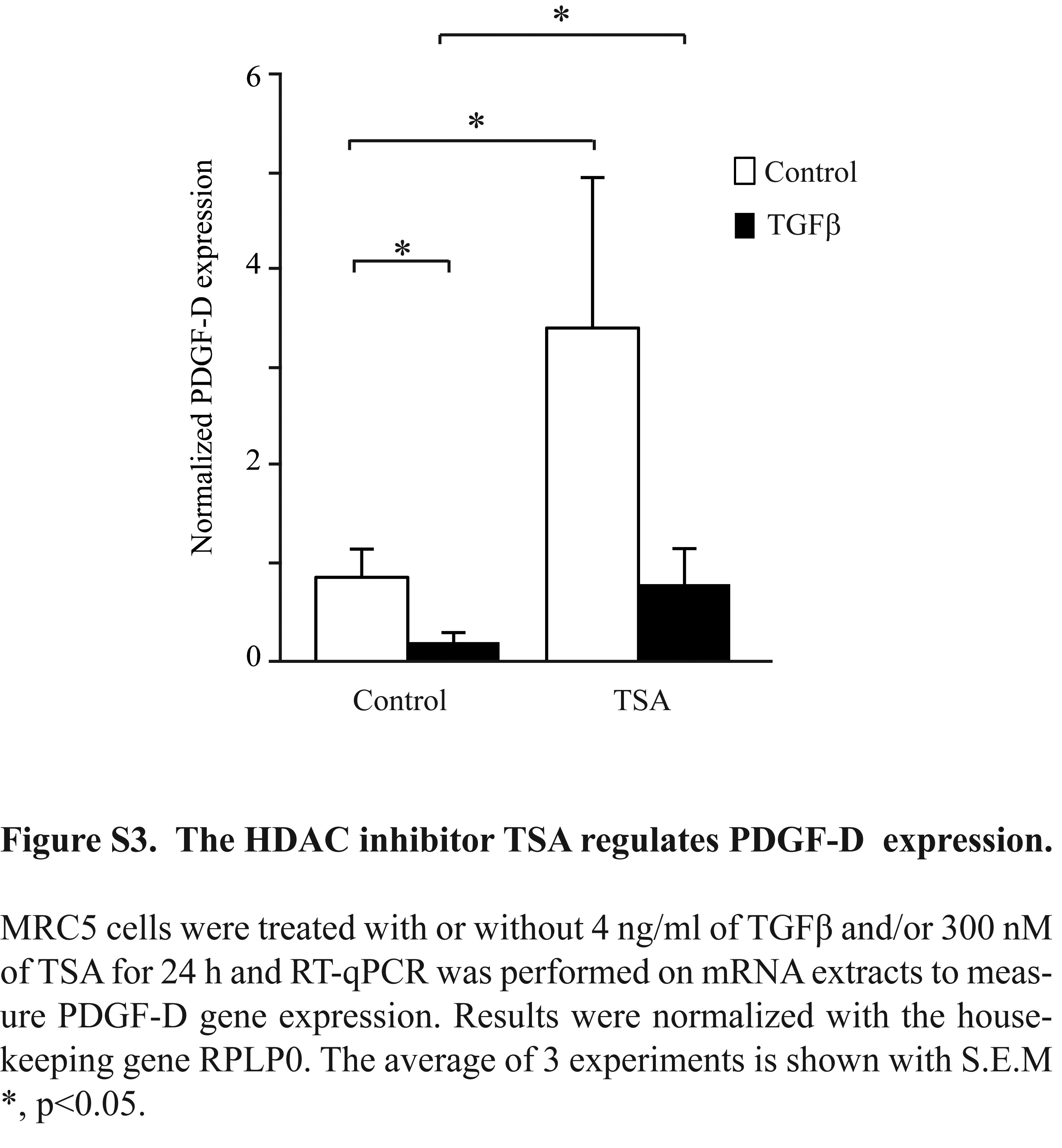

Supplement: Figure S3 — The HDAC inhibitor TSA regulates PDGF-D expression. (TIF) [file pone.0108656.s003.tif]
