# Supplementary material for: PDGF-D Expression Is Down-Regulated by TGFβ in Fibroblasts
Source: PLoS One. 2014 Oct 3;9(10):e108656. doi: 10.1371/journal.pone.0108656 (PMC4184810; doi:10.1371/journal.pone.0108656)
Supplement: Table S1 — q-PCR primer sequences. (DOCX) [file pone.0108656.s004.docx]

**Table S1**

| q-PCR primer sequences | |
| --- | --- |
|  | |
| human Collagen1A1 | CACCTACAGCGTCACTGTCGAT  TGGTTTTGTATTCAATCACGTTCTTG |
| human RPLPO | TCGACAATGGCAGCATCTAC  ATCCGTCTCCACAGACAAGG |
| human PDGF-D | GTGGAGGAAATTGTGGCTGT  CGTTCATGGTGATCCAACTG |
| human PDGF-C | TTCTTGGCAAGGCTTTTGTT  TGCTTGGGACACATTGACAT |
| human SMAD4 | TAGACAGAGAAGCTGGGCGT  CGATGACACTGACGCAAATC |
| mouse Actin | CTCTGGCTCCTAGCACCATGAAG  GCT-GGA-AGG-TGG-ACA-GTG-AG |
| mouse PDGF-D | CTGGACAAAACTGTCGCAGA  GACTGCATTGGTCAGCTTCA |
| mouse PDGF-C | GTGGAGGAAATTGTGCCTGT  TCCAGAGCCACATCAGTGAG |
